# A single-center external validation and refinement of the 9th TNM staging system for thymic epithelial tumors: a scheme enhancing prognostic stratification

**DOI:** 10.3389/fonc.2026.1879433

**Published:** 2026-08-03

**Authors:** Ke Zhao, Xiaoyun Zhou, Lei Liu, Jiaqi Zhang, Guige Wang, Xuehan Gao, Libing Yang, Chao Guo, Cheng Huang, Yeye Chen, Shanqing Li

**Affiliations:** Department of Thoracic Surgery, Peking Union Medical College Hospital, Chinese Academy of Medical Science and Peking Union Medical College, Beijing, China

**Keywords:** thymic epithelial tumor, TNM staging, prognosis, external validation, surgical treatment

## Abstract

**Objective:**

This study aimed to perform a single-center external validation of the recently adopted 9th edition TNM staging system for thymic epithelial tumors (TETs) and to propose a refined scheme addressing its prognostic stratification anomalies.

**Methods:**

We conducted a retrospective cohort study of 540 patients with TETs treated at a single center from 2015 to 2021. All cases were rigorously re-staged according to 9th edition TNM criteria. The prognostic discrimination for overall survival (OS) and progression-free survival (PFS) of 9th edition TNM system was evaluated. Based on identified anomalies, a refined staging scheme was proposed.

**Results:**

The 9th edition TNM system demonstrated overall prognostic capability. However, significant stratification anomalies were observed: stage IIIA patients had paradoxically better OS and PFS than stage II patients, and the long-term survival curve of stage IIIB crossed below that of stage IV. Based on the extent of anatomical invasion, surgical complexity, and survival differences, we propose: (1) downgrading left brachiocephalic vein invasion to T2; (2) merging other T3/T4 lesions into a new T3 category (overall stage IIIA); (3) defining TanyN1M0 as stage IIIB; and (4) defining TanyN2M0 as stage IVA. Application of this refined scheme resulted in statistically significant, monotonically deteriorating trends for both OS and PFS across successive stages (P < 0.001), with markedly improved logical consistency.

**Conclusion:**

This single-center large-scale external validation reveals significant prognostic paradoxes in the 9th edition TNM system for TETs. The proposed refinement scheme effectively optimizes logical consistency and prognostic discrimination, offering potential implications for perioperative therapy planning.

## 1 Introduction

Thymic epithelial tumors (TETs) are a group of rare mediastinal neoplasms with highly heterogeneous clinical behaviors, ranging from indolent growth to aggressive malignancy (1). Complete surgical resection remains the primary curative treatment for localized disease (2–4). Given the typically protracted clinical course and slow progression of TETs, long-term follow-up is crucial for accurately evaluating patient outcomes and treatment efficacy.

A standardized staging system is fundamental for guiding clinical management, predicting prognosis, and facilitating academic communication. This need was ultimately addressed by the development of the TNM staging system by the International Association for the Study of Lung Cancer (IASLC) and the International Thymic Malignancy Interest Group (ITMIG), which was incorporated into the 8th edition of the American Joint Committee on Cancer (AJCC) Cancer Staging Manual (5). While this system has demonstrated a robust ability to stratify prognosis in clinical application, its long-term stability and the precise definition of certain subgroups require further validation with extended follow-up data (5–8).

Building on the analysis of larger global databases, the 9th edition TNM staging system (AJCC Cancer Staging Manual, 9th Edition, 2023) introduced several significant updates and revisions for TETs (9, 10). Key refinement is a new subclassification of the T category which aimed at more precisely correlating the anatomical extent of disease with patient prognosis. However, we have observed that the 9th edition TNM staging system still demonstrates suboptimal performance in certain specific cases. Therefore, this study aims to conduct a retrospective analysis of a treated thymic epithelial tumor cohort from our institution with long-term follow-up. By rigorously re-staging all cases according to the 9th edition TNM criteria, we will evaluate the prognostic value of this new staging system for overall survival (OS) and progression-free survival (PFS) in real-world settings, thereby providing recommendations for the ongoing refinement of this staging system.

## 2 Methods

### 2.1 Study design and patient cohort

This study was a single-center, retrospective cohort study. We consecutively enrolled patients with TETs who received at least one therapeutic intervention (including surgery, chemotherapy, radiotherapy, etc.) at Peking Union Medical College Hospital (PUMCH) between January 1, 2015, and December 31, 2021. This study was approved by the Institutional Review Board (IRB) of PUMCH (Approval No. IRB- K25C3061). The requirement for written informed consent was waived by the IRB of PUMCH.

### 2.2 Inclusion and exclusion criteria

Inclusion Criteria: (1) Age ≥ 18 years; (2) Pathologically confirmed thymic epithelial tumor with a standardized World Health Organization (WHO) histological classification, independently reviewed by at least two pathologists; (3) Availability of complete and traceable clinical data, including detailed medical history, imaging studies (chest CT/MRI), treatment records, and follow-up information; (4) Sufficient data to allow accurate staging according to the 9th edition AJCC TNM staging system.

Exclusion Criteria: (1) Patients who died during the perioperative period; (2) Patients without pathological confirmation; (3) Patients with severely missing clinical data that could compromise reliable analysis; (4) Patients whose tumor extent could not be clearly assessed for accurate 9th edition TNM staging; (5) Patients with complete loss to follow-up.

### 2.3 Data collection and variable definitions

Data were extracted from the hospital’s electronic medical records and medical record archives. The collected variables included:

Independent Variables: tumor stage, age, sex, WHO histological type, clinical symptoms, surgical resection status, and adjuvant therapy.

Pathological staging was used whenever possible. All patients had pathological confirmation of the primary tumor or metastatic lesion. Lymph node status was pathologically confirmed in 210 patients (38.9%); for the remaining low-risk patients, N1 status was assessed by preoperative imaging (PET/CT or chest CT). After excluding M1 patients, 195 patients were staged clinically.

Dependent Variables: The primary endpoints were OS and PFS. OS was defined as the time from the date of pathological diagnosis to death from any cause or the last follow-up. PFS was defined as the time from pathological diagnosis to the first documented disease recurrence, progression, or death from any cause. Recurrence and progression were assessed by contrast enhanced chest CT performed every 6–12 months during follow up, with PET/CT performed when clinically indicated. Whenever clinically feasible, pathological confirmation of suspected recurrence or progression was obtained by biopsy. Disease progression was defined according to RECIST criteria (version 1.1) (11) for measurable disease.

All enrolled cases were rigorously re-staged according to the 8th edition AJCC TNM staging system, and the 9th edition AJCC TNM staging system, leading to the proposal of a novel refined 9th TNM staging proposal for TETs. Restaging was performed independently by two thoracic surgeons based on all available clinical, imaging, operative, and pathological records. The reviewers were blinded to patient outcomes. In cases of disagreement, consensus was reached through discussion with a third senior reviewer. Both clinical and pathological TNM were recorded; pathological staging was used as the primary reference for patients who underwent surgical resection. Survival status and disease progression were confirmed via outpatient reviews and telephone interviews, with the last follow-up date being June 30, 2025.

### 2.4 Statistical analysis

Categorical variables were described using frequencies and percentages (n, %), and continuous variables were presented as mean ± standard deviation or median with interquartile range (IQR), as appropriate. Inter-group comparisons were performed using Student’s t-test (for continuous variables) or Chi-square/Fisher’s exact test (for categorical variables). Survival curves were plotted using the Kaplan-Meier method, and differences among staging subgroups were compared using the Log-rank test. For multivariable analysis, separate Cox proportional hazards regression models were constructed for the original TNM9 and the refined TNM9 staging systems. Variables with p < 0.10 in univariable analysis were entered into multivariable models using forward stepwise selection. The proportional hazards assumption was tested using Schoenfeld residuals; all models were estimated with robust standard errors. Model performance was compared using the C index, Akaike information criterion (AIC), and time dependent area under the ROC curve (AUC) at 24, 60, and 120 months. Missing data were handled by complete case analysis. A two-sided p < 0.05 was considered statistically significant. All statistical analyses were performed using R software (version 4.5.2).

## 3 Results

### 3.1 Patient cohort and baseline characteristics

Using the inclusion and exclusion criteria described above, a total of 540 patients were finally enrolled. The clinicopathologic characteristics are detailed in **Table 1**. Complete loss to follow-up means that, at the end of the study, the investigators could not ascertain whether the patient was alive, dead, or had tumor progression.

**Table 1:** Demographic and clinicopathological characteristics of the study patients.

Variables	Values
Size (cm, mean ± SD, md* = 13)	5.1(2.5)
Size group (%, NA = 13)	
≤5.0cm	322(61.1)
>5.0cm	205(38.9)
Age (years, mean ± SD, md = 0)	51.9(12.0)
Age group (%, NA = 0)	
≤45 years	144(26.7)
≤60 years and >45years	233(43.1)
>60 years	163(30.2)
Sex (%, NA = 0)	
Female	276(51.1)
Male	264(48.9)
Pathologic type (%, md = 0)	
Type A	43(8.0)
Type AB	144(26.7)
Type B1	66(12.2)
Type B2	98(18.2)
Type B3	41(7.6)
Multiple histological subtypes coexisting	45(8.3)
Other atypical types of thymoma	19(3.5)
Carcinoma	80(14.8)
Neuroendocrine tumor	4(0.7)
Comorbidity (%, md = 0)	
Without comorbidity	389(72.0)
With Myasthenia Gravis	126(23.4)
With other autoimmune diseases	25(4.6)
Surgery (%, md = 0)	
No	24(4.4)
Yes	516(95.6)
Surgery approach (%, md = 26)	
Video-assisted thoracoscopic surgery	365(71.0)
Subxiphoid video-assisted thoracoscopic surgery	22(4.3)
Anterior cervical incision	3(0.6)
Median sternotomy	112(21.8)
Lateral thoracotomy	5(1.0)
VATS conversion to thoracotomy	7(1.3)
R0 resection (%, md = 16)	
Yes	490(93.5)
No	34(6.5)
Perioperative radiotherapy (%, md = 1)	
Yes	210(39.0)
No	329(61.0)
Perioperative chemotherapy (%, md = 1)	
Yes	78(14.5)
No	461(85.5)

* md: missing data.

### 3.2 Survival analysis results

**Figure 1:**
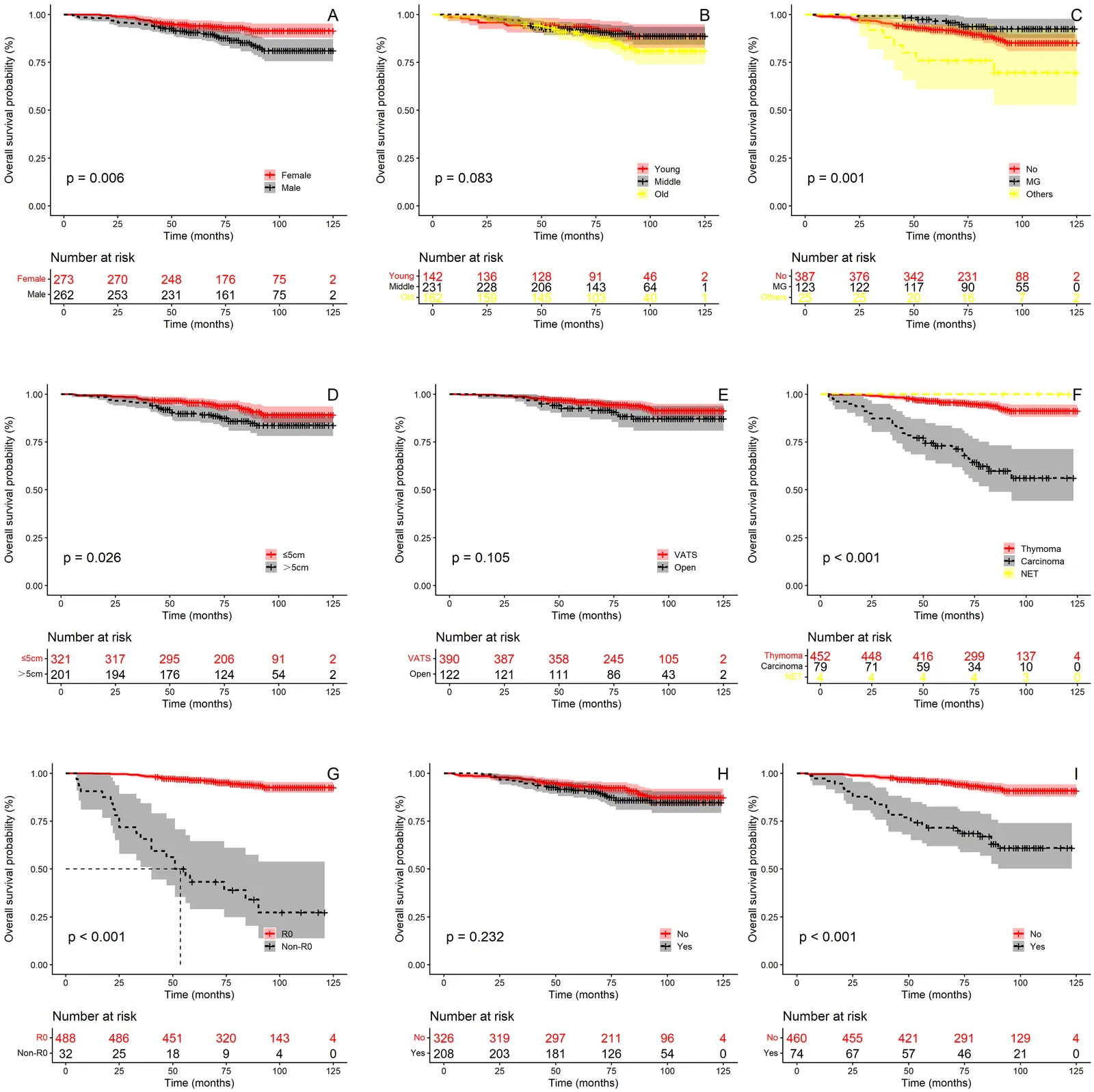
Kaplan-Meier survival curves for overall survival **(OS)** stratified by various factors in the cohort. **(A)** Sex; **(B)** Age group; **(C)** Comorbidity status; **(D)** Tumor diameter group; **(E)** Surgical approach; **(F)** Histological type; **(G)** R0 resection status; **(H)** Perioperative radiotherapy; **(I)** Perioperative chemotherapy.

**Figure 2:**
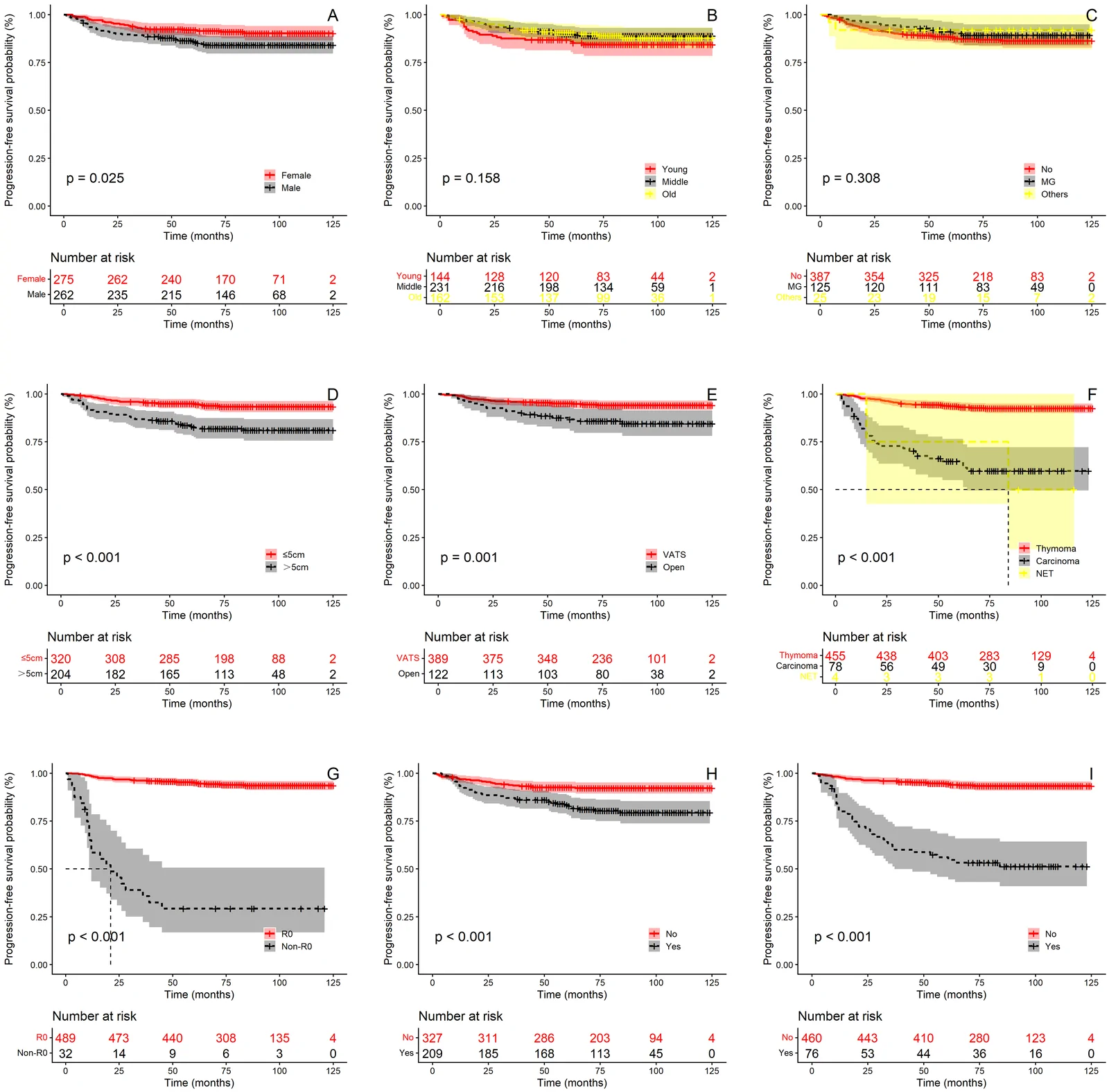
Kaplan-Meier survival curves for progression-free survival (PFS) stratified by various factors in the cohort. **(A)** Sex; **(B)** Age group; **(C)** Comorbidity status; **(D)** Tumor diameter group; **(E)** Surgical approach; **(F)** Histological type; **(G)** R0 resection status; **(H)** Perioperative radiotherapy; **(I)** Perioperative chemotherapy.

The median follow up duration for the entire cohort was 85 months (IQR: 66–103). A total of 59 deaths and 65 progression events were recorded during the follow up period. The investigators first conducted OS and PFS analyses for all patients stratified by different subgroups; the results are as follows (**Figures 1**, **2**).

Factors showing a significant impact on OS were sex, comorbidity, maximum tumor diameter category, histological subtype, achievement of R0 resection, and receipt of perioperative chemotherapy. No significant influence was observed for age group, surgical approach, or perioperative radiotherapy. For PFS, significant factors included sex, maximum tumor-diameter category, histological subtype, surgical approach, R0 resection, perioperative radiotherapy, and perioperative chemotherapy, whereas age group and comorbidity had no significant effect.

### 3.3 Validation and limitations of the 9th edition staging system in the present cohort

**Figure 3:**
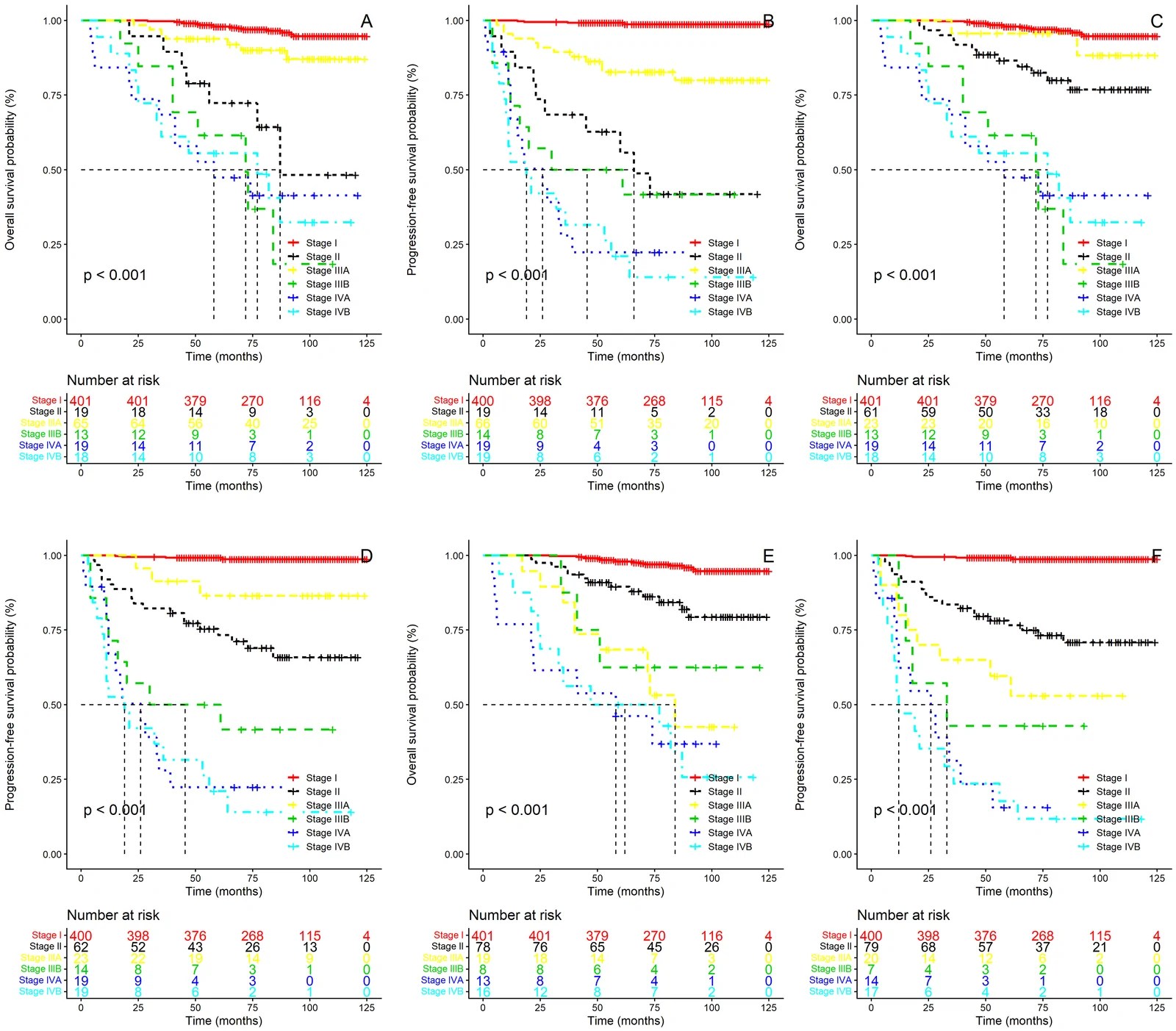
Kaplan-Meier survival curves for overall survival (OS) and progression-free survival (PFS) stratified by each stage of the 8th, 9th, and refined 9th edition TNM staging system. **(A, B)** for 8th staging system; **(C, D)** for 9th staging system; **(E, F)** for refined 9th staging system.

**Figure 4:**
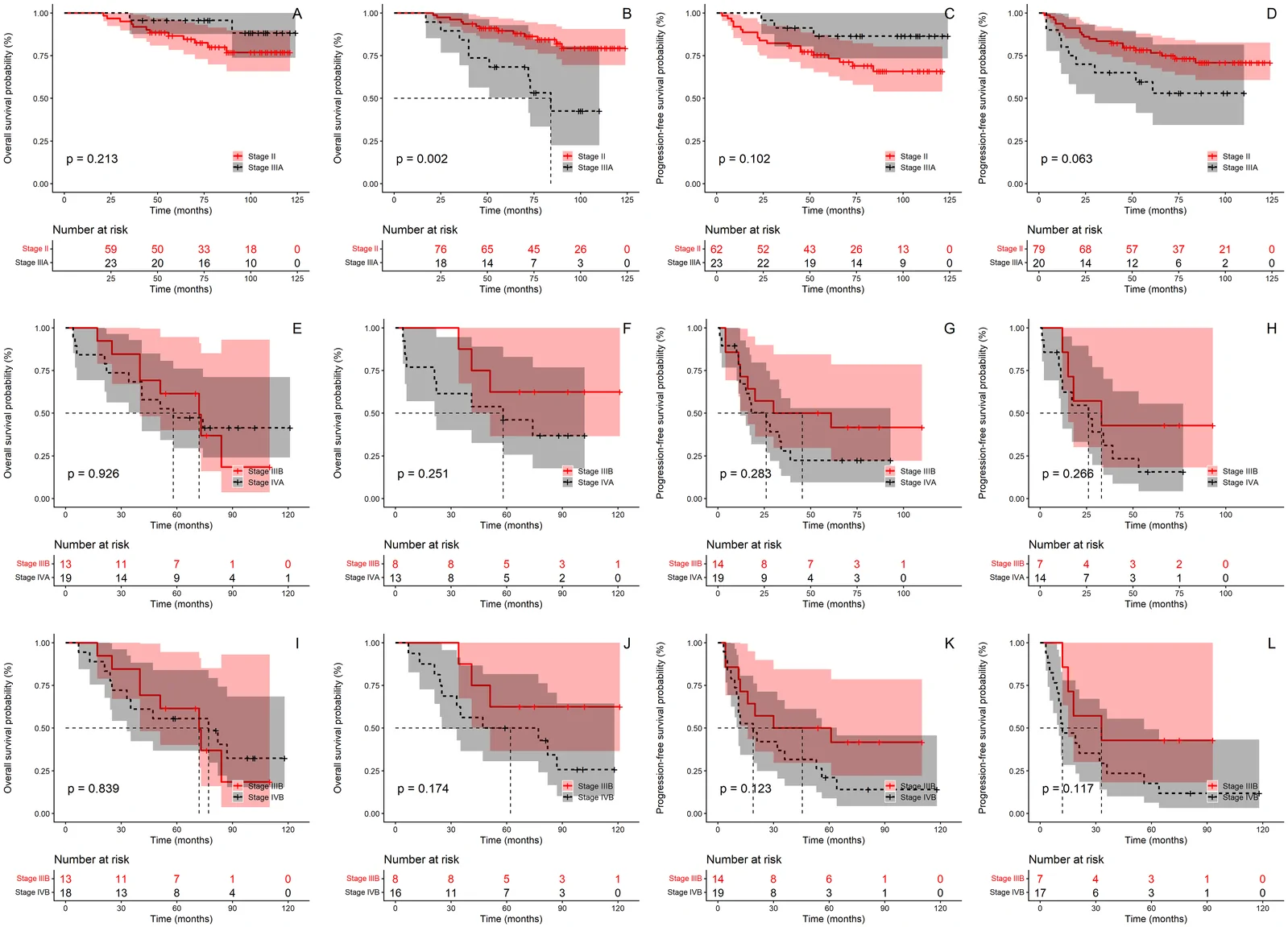
Kaplan-Meier curves for overall survival (OS) and progression-free survival (PFS) in subgroups exhibiting paradoxical survival under the original 9th staging system versus their counterparts under the refined 9th staging system. **(A, C, E, G, I, K)** for original 9th staging system; **(B, D, F, H, J, L)** for refined 9th staging system.

Survival analyses showed that the 9th-edition TNM classification predicted prognosis in our cohort (**Figure 3**); however, outcomes were paradoxically better for stage IIIA than for stage II patients, with fewer deaths and recurrences recorded in the IIIA subgroup (**Figure 4**). Moreover, after 6 years of follow-up the overall-survival curve of stage IIIB patients fell below that of stage IV patients (**Figure 4**). These findings indicate that the current definitions of 9th TNM staging system still merit refinement.

### 3.4 Proposed refinements for the 9th edition of the TNM staging system

**Figure 5:**
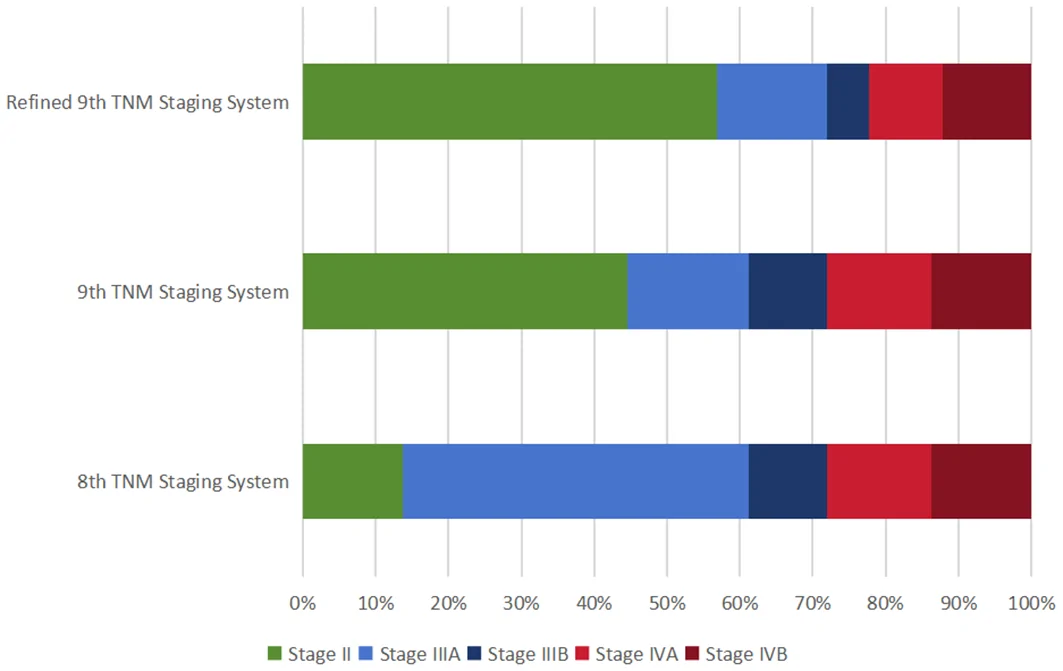
Distribution of patients by stage according to different staging systems.

We therefore propose the following modifications: (1) classify invasion of the left brachiocephalic vein as T2; (2) merge the remaining original T3 and T4 categories into a new T3 category, with an overall stage of IIIA; (3) designate any T, N1, M0 disease as stage IIIB; and (4) assign any T, N2, M0 tumor to stage IVA. The rationale for these adjustments will be elaborated in the discussion. After re-classifying patients according to the revised 9th edition TNM scheme, the number of patients in both stage III and stage IV showed a moderate decrease (**Figure 5**, **Table 2**), and the investigators repeated survival analyses. Stage I is not shown in **Figure 5** because our proposed refinements primarily affect stages II, III, and IV, while stage I remained unchanged. Outcomes deteriorated in a stepwise fashion across stages for both OS and PFS (**Figure 3**), with the OS difference between stage II and stage IIIA achieving statistical significance (**Figure 4**).

**Table 2:** The detailed TNM distribution table of all patients.

Variables	TNM8	TNM9	Modified
T category – n (%)			
T1	411(76.1)	411(76.1)	411(76.1)
T2	20(3.7)	63(11.7)	82(15.2)
T3	81(15.0)	38(7.0)	37(6.8)
T4	18(3.3)	18(3.3)	N/A
TX	10(1.9)	10(1.9)	10(1.9)
N category – n (%)			
N0	513(95.0)	513(95.0)	513(95.0)
N1	13(2.4)	13(2.4)	13(2.4)
N2	3(0.6)	3(0.6)	3(0.6)
NX	11(2.0)	11(2.0)	11(2.0)
M category – n (%)			
M0	525(97.2)	525(97.2)	525(97.2)
M1	15(2.8)	15(2.8)	15(2.8)
T3 substructures* – n (% of T3)			
Lung	20(24.7)	N/A	N/A
Phrenic nerve	31(38.3)	N/A	N/A
Left brachiocephalic vein	19(23.5)	19(50.0)	N/A
Right brachiocephalic vein	5(6.2)	5(13.2)	5(13.5)
Superior vena cava	7(8.6)	7(18.4)	8(21.6)
Chest wall	2(2.5)	2(5.3)	2(5.4)
Hilar pulmonary vessels	8(9.9)	8(21.1)	9(24.3)

*: Patients may have multiple T3 substructures; percentages are calculated within each staging system’s T3 subgroup.

To formally assess the stepwise prognostic pattern across stages, we performed a trend test by treating stage as a continuous variable in a univariable Cox regression model for the refined TNM9 staging system. The test confirmed a significant linear trend across stages for both OS (HR = 1.94, 95% CI: 1.72–2.19, χ² = 85.71, df = 1, p < 0.001) and PFS (HR = 2.30, 95% CI: 2.05–2.59, χ² = 153.6, df = 1, p < 0.001).

### 3.5 Univariate and multivariate Cox regression analysis

To compare the prognostic performance of the original TNM9 and refined TNM9 staging systems, we constructed separate multivariable Cox models for each. The refined TNM9 system performed comparably to the original TNM9 in terms of model fit and discriminatory ability, while demonstrating improved logical consistency and resolving the paradoxical survival inversions observed in the original system.

For the original TNM9 model, independent predictors of OS included: age, sex, histological type, R0 resection status, and original TNM9 stage. Independent predictors of PFS included: sex, histological type, R0 resection status, radiotherapy, chemotherapy and original TNM9 stage. For the refined TNM9 model, independent predictors of OS included: age, sex, histological type, R0 resection status and refined TNM9 stage. Independent predictors of PFS included: sex, histological type, R0 resection status, radiotherapy, chemotherapy and original TNM9 stage. Detailed results are presented in **Supplementary Tables 1–4**.

## 4 Discussion

This study conducted an independent single-center external validation of the 9th edition AJCC TNM staging system through long-term follow-up and rigorous re-staging of a large single-center cohort of TETs (9). Our findings confirm that the new edition overall possesses prognostic predictive capability, but simultaneously reveal significant prognostic stratification anomalies within certain specific subgroups. These are primarily manifested as the “crossover phenomenon,” where stage IIIA patients exhibited better outcomes than stage II patients, and the long-term survival of stage IIIB patients was inferior to that of stage IV patients. Based on this empirical data, we proposed a set of specific revisions, and preliminary validation suggests that this proposal effectively enhances the logical consistency and prognostic stratification ability of the staging system.

As is well known, the TNM staging system classifies tumors into stages I through IV to reflect the degree of anatomical spread (12). Typically, tumors confined to the organ of origin are defined as stage I and are managed primarily with surgical intervention. With the presence of local invasion or regional lymph node metastasis, the stage advances to II or III, where a comprehensive treatment strategy centered on surgery is often adopted. In cases of distant metastasis, designated as stage IV, systemic therapy becomes the mainstay of treatment. This stratification logic has been established as a consensus in numerous solid tumors and offers valuable insights for staging research in TETs (13–16).

Based on the aforementioned principles and in light of the existing limitations of the 9th edition staging system, we propose the following revisions: 1) Reclassify invasion of the left brachiocephalic vein as T2; and 2) Merge the remaining original T3 and T4 categories into a new T3 category, corresponding to an overall stage of IIIA. These adjustments are grounded in three key considerations. First, patients with left brachiocephalic vein invasion generally exhibit favorable overall prognosis and constituted a substantial proportion (17/23, 73.9%) of the cases previously classified as stage IIIA. Retaining this group within the T3 category could result in a statistical distortion where the OS data for stage IIIA appears paradoxically better than that for stage II. This observation is consistent with earlier literature (17, 18). Second, from a surgical perspective, the technical management of the left brachiocephalic vein is more straightforward compared to structures such as the superior vena cava, hilar vessels, aorta, and chest wall. Whether employing venoplasty, bypass, or division, achieving an R0 resection is generally more feasible, which likely constitutes a significant reason for the more favorable prognosis in these cases (4, 17). Furthermore, from an anatomical standpoint, the pericardium, bilateral phrenic nerves, and left brachiocephalic vein collectively form the natural boundaries of the thymus and can be regarded as the primary barrier against tumor extension (19). Thus, it is anatomically rational to consider invasion of these structures as belonging to the same severity tier. In contrast, invasion beyond these structures not only indicates more advanced disease but also presents significantly greater surgical challenges, often necessitating multimodal treatment approaches (20, 21); hence, such cases should appropriately remain classified as Stage III. Given that the original T3 and T4 patients in our cohort demonstrated comparable survival outcomes and shared similar therapeutic principles and surgical complexity, merging them into a new T3 category (overall stage IIIA) serves to streamline the staging system while preserving its clinical utility.

Furthermore, survival outcomes within this cohort demonstrated that patients with TanyN1M0 disease (5 alive patients and 3 dead patients) exhibited more favorable OS compared to other patients originally classified as stage IV (10 alive patients and 19 dead patients). Although this difference did not reach statistical significance (p=0.153), the observed trend toward a survival advantage holds clinical relevance. This finding is consistent with data from the ITMIG cohort, which also indicates a better prognosis for patients with N1 metastasis (10). From the perspective of local control, metastatic foci in N1 lymph nodes are more amenable to management through surgery compared to distant metastases (22, 23). Based on these reasons, the investigators recommend downstaging TanyN1M0 cases to stage IIIB. However, given the very small sample size and the non-significant comparison, we emphasize that this finding should be considered exploratory and hypothesis-generating, and further validation in larger cohorts is required before any formal staging modification for N1 disease can be recommended.

This cohort contained only 2 patients with TanyN2M0 disease which prevents meaningful statistical analysis, N2 metastasis refers to tumor spread to deep intrathoracic or cervical lymph nodes (13). From a biological behavior perspective, N2 metastasis indicates involvement of more distant lymph node regions, generally carrying a higher tumor burden and greater metastatic risk compared to N1 metastasis. Nevertheless, it remains fundamentally distinct from M1 disease characterized by distant hematogenous metastasis. Therefore, from a conceptual and anatomical perspective, the investigators propose classifying TanyN2M0 disease as stage IVA, positioning it between stage IIIB (TanyN1M0) and stage IVB (TanyNanyM1b). As shown in **Figure 3** and **Figure 4**, application of this revised staging scheme resulted in statistically significant, monotonically decreasing trends in both OS and PFS across successive stages, thereby confirming its clinical validity and prognostic utility.

We believe that this refined scheme may offer certain practical value for key phases of surgical practice in thymic tumors. Preoperatively, downgrading left brachiocephalic vein invasion to T2 allows for a more precise assessment of surgical complexity and the likelihood of R0 resection. Intraoperatively, consolidating other locally advanced invasions into a new T3 category (stage IIIA) recognizes their shared high-risk anatomical nature, necessitating preparedness for complex maneuvers and adherence to en-bloc resection; concurrently, classifying N1 disease as stage IIIB highlights the importance of systematic lymph node sampling for accurate staging. Postoperatively, the refined scheme eliminates the paradoxical survival inversion present in the original staging, providing a logically consistent foundation for deciding on adjuvant therapies (e.g., radiotherapy or chemotherapy) based on more accurate staging.

Currently, external validation studies on the 9th edition TNM staging system for TETs remain scarce. Existing research has primarily focused on the prognostic significance of tumor size using the 5-cm cutoff. For example, Hashinokuchi et al. (24) reported that patients with thymoma and tumors ≤5 cm had significantly longer OS than those with tumors >5 cm (median OS 193.2 months vs. 161.4 months, P < 0.0001; HR 0.72, 95% CI 0.64–0.82). Multivariate analysis confirmed tumor size as an independent prognostic factor for OS (P < 0.001), although this association was not significant in thymic carcinoma (p = 0.099). In contrast, Chiappetta et al. (25) suggested that tumor size >5 cm is an independent risk factor for OS in all TETs (p < 0.001, HR 2.16, 95% CI 1.32–3.50). However, neither study performed a dedicated subgroup analysis specifically within the T1 category. Other studies have also indicated that tumor size affects tumor invasiveness; a maximum tumor diameter exceeding 6 cm is associated with a higher risk of local invasion (26). However, that study did not discuss the impact of tumor size on prognosis. In our cohort, tumor size >5 cm did not reach statistical significance in multivariate analysis, and its impact on OS and PFS was even less pronounced within the T1 subgroup. We attribute this finding mainly to insufficient sample size and resultant low statistical power. It is noteworthy that even when statistical significance is achieved in larger studies, the actual survival difference between the T1a and T1b subgroups may be clinically minimal.

In comparing the prognostic performance between the original TNM9 and the refined TNM9 staging systems, our multivariable models yielded comparable results across multiple metrics. For OS, the refined model showed an AIC of 457.19 (vs. 457.15 for the original) and a C-index of 0.889 (95% CI: 0.801–0.975) (vs. 0.894, 95% CI: 0.807–0.981), with time-dependent AUCs ranging from 0.901 to 0.971 across 24, 60, and 120 months (vs. 0.905–0.968 for the original). For PFS, the refined model demonstrated an AIC of 401.24 (vs. 400.45 for the original), a C-index of 0.872 (95% CI: 0.778–0.967) (vs. 0.874, 95% CI: 0.779–0.969), and time-dependent AUCs of 0.815–0.990 (vs. 0.813–0.992 for the original).

Notably, the proportional hazards assumption was marginally violated for the original TNM9 OS model (global p = 0.048) but satisfied for the refined TNM9 OS model (global p = 0.059). This suggests that the refined system may provide more stable risk estimates over time. Furthermore, the refined staging corrected the paradoxical stage inversions observed in the original system—specifically, the better outcomes of stage IIIA versus stage II, and the crossing of stage IIIB below stage IV after prolonged follow-up—without sacrificing prognostic discrimination. This combination of statistical robustness and clinical logical consistency supports the potential utility of the refined scheme in real-world practice. Nevertheless, we acknowledge that the performance differences between the two systems are modest, and the proposed refinements—particularly the N-related changes—require validation in larger, multi-center cohorts.

The multivariate analysis in this study revealed that after adjusting for TNM stage, WHO histologic type were independent predictors for OS and PFS. This finding suggests that the tumor’s histology may influence long-term patient outcomes beyond the initial anatomical extent of disease. This perspective is also consistent with multiple previous studies (27–29). This finding suggests the potential need to develop a TNMG staging system—similar to that used for esophageal cancer (30)—to guide the management of TETs with greater precision.

This study has several limitations. First, as a retrospective study, it is inevitably subject to selection bias. Second, despite the implementation of strict quality control measures, the potential for missing certain historical data cannot be entirely eliminated. Furthermore, while the revision scheme we proposed performed well within this cohort, its generalizability requires further validation in prospective, multi-center, larger-scale independent cohorts. Finally, the adjustment of a staging system is a complex and rigorous process that necessitates international consensus; our proposal aims to provide empirical evidence to inform this ongoing academic discourse.

To the best of our knowledge, this represents the largest external validation cohort to date for the 9th edition TNM staging system in TETs. In summary, this study validates the overall applicability of the 9th edition AJCC TNM staging system in TETs, while also revealing its internal stratification deficiencies. The proposed revision scheme preliminarily demonstrates its potential to improve the logical consistency and precision of prognostic stratification. We recommend that future revisions of the staging system should fully consider the prognostic weight of specific vascular invasions, simplify overlapping T categories, and place significant emphasis on the staging implications of lymph node metastasis.

## Data Availability

The datasets generated and/or analyzed during the current study are available from the corresponding author upon reasonable request. Requests to access the datasets should be directed to Chen Yeye, chenyeye@pumch.cn.
