# Supplementary material for: A single-center external validation and refinement of the 9th TNM staging system for thymic epithelial tumors: a scheme enhancing prognostic stratification
Source: Front Oncol. 2026 Aug 3;16:1879433. doi: 10.3389/fonc.2026.1879433 (PMC13494866; doi:10.3389/fonc.2026.1879433)
Supplement: Supplementary file 1 [file SupplementaryFile1.docx]

| Variables | Univariate analyses | | Multivariate analyses | |
| --- | --- | --- | --- | --- |
|  | HR (95% CI) | P value | HR (95% CI) | P value |
| Age (per 1‑year increase) | 1.03 (1.01-1.05) | **0.015** | 1.04 (1.01-1.07) | **0.018** |
| Age group |  | 0.241 |  |  |
| 18 yeras-44 years | 1 |  |  |  |
| 45 years-59 years | 1.06 (0.53-2.10) | 0.874 |  |  |
| ≥60 years | 1.63 (0.83-3.20) | 0.156 |  |  |
| Sex |  |  |  |  |
| Female | 1 |  | 1 |  |
| Male | 2.09 (1.22-3.58) | **0.008** | 3.05 (1.53-6.10) | **0.002** |
| Size (per 1cm increase) | 1.09 (1.00-1.20) | **0.046** | 0.88 (0.72-1.09) | 0.252 |
| Size group |  |  |  |  |
| ≤5cm | 1 |  | 1 |  |
| ＞5cm | 1.83 (1.07-3.14) | **0.028** | 2.24 (0.80-6.26) | 0.123 |
| Comorbidity |  | **0.011** |  | 0.741 |
| No Comorbidity | 1 |  | 1 |  |
| Myasthenia Gravis | 0.50 (0.24-1.07) | 0.073 | 0.67 (0.23-1.92) | 0.451 |
| Other autoimmune diseases | 2.49 (1.12-5.53) | **0.025** | 3.63 (1.08-12.16) | **0.038** |
| Pathologic type |  | **<0.001** |  | **0.010** |
| Thymoma | 1 |  | 1 |  |
| Thymic cancer | 7.2 (4.32-12.03) | **<0.001** | 2.29 (1.03-5.11) | **0.043** |
| Thymic neuroendocrine carcinoma | 0.00 (0.00-Inf) | 0.997 | 0.00 (0.00-Inf) | 0.997 |
| R0 resection status |  |  |  |  |
| Yes | 1 |  | 1 |  |
| No | 18.67 (10.56-33.04) | **<0.001** | 5.72 (1.91-17.12) | **0.002** |
| Surgery approach |  |  |  |  |
| Minimally invasive | 1 |  |  |  |
| Open surgery | 1.71 (0.89-3.29) | 0.109 |  |  |
| TNM9 staging system |  | **<0.001** |  | **0.001** |
| I | 1 |  | 1 |  |
| II | 5.81 (2.72-12.41) | **<0.001** | 4.25 (1.76-10.29) | **0.001** |
| IIIA | 2.28 (0.52-9.99) | 0.273 | 1.96 (0.41-9.27) | 0.398 |
| IIIB | 25.57 (10.77-60.71) | **<0.001** | 1.68 (0.33-8.52) | 0.534 |
| IVA | 24.02 (11.02-52.37) | **<0.001** | 7.60 (1.91-30.23) | **0.004** |
| IVB | 24.46 (11.22-53.32) | **<0.001** | 13.65 (3.68-50.67) | **<0.001** |
| Radiotherapy |  |  |  |  |
| No | 1 |  |  |  |
| Yes | 1.37 (0.82-2.28) | 0.234 |  |  |
| Chemotherapy |  |  |  |  |
| No | 1 |  | 1 |  |
| Yes | 5.73 (3.43-9.56) | **<0.001** | 0.55 (0.21-1.44) | 0.223 |

**Supplementary Table 1 Multivariable Cox regression analysis of overall survival incorporating the original TNM9 staging system^*^**

**Model performance metrics: AIC^#^ = 457.15, likelihood ratio test P < 0.001, C‑index = 0.894 (95% CI^#^: 0.807–0.981), time‑dependent AUCs at 24, 60, and 120 months were 0.968, 0.909, and 0.905, respectively, and PH^#^ assumption test P = 0.048.*

*#Abbreviations: AIC, Akaike information criterion; CI, confidence interval; AUC, area under curve; PH, proportional hazards.*

| Variables | Univariate analyses | | Multivariate analyses | |
| --- | --- | --- | --- | --- |
|  | HR (95% CI) | P value | HR (95% CI) | P value |
| Age (per 1‑year increase) | 1.03 (1.01-1.05) | **0.015** | 1.04 (1.01-1.07) | **0.009** |
| Age group |  | 0.241 |  |  |
| 18 yeras-44 years | 1 |  |  |  |
| 45 years-59 years | 1.06 (0.53-2.10) | 0.874 |  |  |
| ≥60 years | 1.63 (0.83-3.20) | 0.156 |  |  |
| Sex |  |  |  |  |
| Female | 1 |  | 1 |  |
| Male | 2.09 (1.22-3.58) | **0.008** | 3.30 (1.61-6.74) | **0.001** |
| Size (per 1cm increase) | 1.09 (1.00-1.20) | **0.046** | 0.86 (0.69-1.08) | 0.195 |
| Size group |  |  |  |  |
| ≤5cm | 1 |  | 1 |  |
| ＞5cm | 1.83 (1.07-3.14) | **0.028** | 2.50 (0.84-7.40) | 0.098 |
| Comorbidity |  | **0.011** |  | 0.741 |
| No Comorbidity | 1 |  | 1 |  |
| Myasthenia Gravis | 0.50 (0.24-1.07) | 0.073 | 0.71 (0.25-2.02) | 0.520 |
| Other autoimmune diseases | 2.49 (1.12-5.53) | **0.025** | 3.34 (1.00-11.18) | 0.050 |
| Pathologic type |  | **<0.001** |  | **0.010** |
| Thymoma | 1 |  | 1 |  |
| Thymic cancer | 7.2 (4.32-12.03) | **<0.001** | 2.43 (1.10-5.33) | **0.027** |
| Thymic neuroendocrine carcinoma | 0.00 (0.00-Inf) | 0.997 | 0.00 (0.00-Inf) | 0.997 |
| R0 resection status |  |  |  |  |
| Yes | 1 |  | 1 |  |
| No | 18.67 (10.56-33.04) | **<0.001** | 4.41 (1.33-14.62) | **0.015** |
| Surgery approach |  |  |  |  |
| Minimally invasive | 1 |  |  |  |
| Open surgery | 1.71 (0.89-3.29) | 0.109 |  |  |
| Refined TNM9 staging system |  | **<0.001** |  | **0.001** |
| I | 1 |  | 1 |  |
| II | 4.76 (2.26-10.00) | **<0.001** | 3.67 (1.58-8.56) | **0.003** |
| IIIA | 17.10 (7.47-39.16) | **<0.001** | 1.91 (0.42-8.66) | 0.400 |
| IIIB | 12.02 (3.48-41.54) | **<0.001** | 4.79 (0.79-28.89) | 0.088 |
| IVA | 28.29 (11.98-66.81) | **<0.001** | 12.91 (2.32-71.99) | **0.004** |
| IVB | 29.18 (13.38-63.65) | **<0.001** | 14.78 (3.94-55.42) | **<0.001** |
| Radiotherapy |  |  |  |  |
| No | 1 |  |  |  |
| Yes | 1.37 (0.82-2.28) | 0.234 |  |  |
| Chemotherapy |  |  |  |  |
| No | 1 |  | 1 |  |
| Yes | 5.73 (3.43-9.56) | **<0.001** | 0.67 (0.24-1.92) | 0.458 |

**Supplementary Table 2 Multivariable Cox regression analysis of overall survival incorporating the refined TNM9 staging system^*^**

**Model performance metrics: AIC^#^ = 457.19, likelihood ratio test P < 0.001, C‑index = 0.889 (95% CI^#^: 0.801–0.975), time‑dependent AUCs at 24, 60, and 120 months were 0.971, 0.901, and 0.914, respectively, and PH^#^ assumption test P = 0.059.*

*#Abbreviations: AIC, Akaike information criterion; CI, confidence interval; AUC, area under curve; PH, proportional hazards.*

| Variables | Univariate analyses | | Multivariate analyses | |
| --- | --- | --- | --- | --- |
|  | HR (95% CI) | P value | HR (95% CI) | P value |
| Age (per 1‑year increase) | 0.99 (0.97-1.01) | 0.316 |  |  |
| Age group |  | 0.241 |  |  |
| 18 yeras-44 years | 1 |  |  |  |
| 45 years-59 years | 1.06 (0.53-2.10) | 0.874 |  |  |
| ≥60 years | 1.63 (0.83-3.20) | 0.156 |  |  |
| Sex |  |  |  |  |
| Female | 1 |  | 1 |  |
| Male | 1.76 (1.07-2.90) | **0.027** | 3.28 (1.53-7.05) | **0.002** |
| Size (per 1cm increase) | 1.19 (1.10-1.28) | **<0.001** | 0.86 (0.68-1.09) | 0.227 |
| Size group |  |  |  |  |
| ≤5cm | 1 |  | 1 |  |
| ＞5cm | 3.08 (1.79-5.31) | **<0.001** | 2.12 (0.71-6.31) | 0.176 |
| Comorbidity |  | **0.011** |  | 0.119 |
| No Comorbidity | 1 |  | 1 |  |
| Myasthenia Gravis | 0.50 (0.24-1.07) | 0.073 | 0.65 (0.22-1.94) | 0.436 |
| Other autoimmune diseases | 2.49 (1.12-5.53) | **0.025** | 7.18 (2.26-22.80) | **0.001** |
| Pathologic type |  | **<0.001** |  | **<0.001** |
| Thymoma | 1 |  | 1 |  |
| Thymic cancer | 7.20 (4.32-12.03) | **<0.001** | 4.97 (1.93-12.80) | **0.001** |
| Thymic neuroendocrine carcinoma | 0.00 (0.00-Inf) | 0.997 | 0.00 (0.00-Inf) | 0.997 |
| R0 resection status |  |  |  |  |
| Yes | 1 |  | 1 |  |
| No | 21.38 (12.17-37.56) | **<0.001** | 4.31 (1.07-17.33) | **0.040** |
| Surgery approach |  |  |  |  |
| Minimally invasive | 1 |  | 1 |  |
| Open surgery | 2.66 (1.42-4.95) | **0.002** | 1.04 (0.41-2.62) | 0.931 |
| TNM9 staging system |  | **<0.001** |  | **0.002** |
| I | 1 |  | 1 |  |
| II | 5.81 (2.72-12.41) | **<0.001** | 6.76 (2.60-17.53) | **<0.001** |
| IIIA | 2.28 (0.52-9.99) | 0.273 | 2.96 (0.59-14.70) | 0.186 |
| IIIB | 25.57 (10.77-60.71) | **<0.001** | 2.42 (0.33-17.66) | 0.384 |
| IVA | 24.02 (11.02-52.37) | **<0.001** | 6.87 (1.39-34.00) | **0.018** |
| IVB | 24.46 (11.22-53.32) | **<0.001** | 12.16 (2.34-63.16) | **0.003** |
| Radiotherapy |  |  |  |  |
| No | 1 |  | 1 |  |
| Yes | 2.64 (1.60-4.36) | **<0.001** | 0.33 (0.14-0.78) | **0.011** |
| Chemotherapy |  |  |  |  |
| No | 1 |  | 1 |  |
| Yes | 9.60 (5.88-15.67) | **<0.001** | 0.32 (0.10-0.99) | **0.047** |

**Supplementary Table 3 Multivariable Cox regression analysis of progression-free survival incorporating the original TNM9 staging system^*^**

**Model performance metrics: AIC^#^ = 400.45, likelihood ratio test P < 0.001, C‑index = 0.874 (95% CI^#^: 0.779–0.969), time‑dependent AUCs at 24, 60, and 120 months were 0.992, 0.889, and 0.813, respectively, and PH^#^ assumption test P = 0.390.*

*#Abbreviations: AIC, Akaike information criterion; CI, confidence interval; AUC, area under curve; PH, proportional hazards.*

| Variables | Univariate analyses | | Multivariate analyses | |
| --- | --- | --- | --- | --- |
|  | HR (95% CI) | P value | HR (95% CI) | P value |
| Age (per 1‑year increase) | 0.99 (0.97-1.01) | 0.316 |  |  |
| Age group |  | 0.241 |  |  |
| 18 yeras-44 years | 1 |  |  |  |
| 45 years-59 years | 1.06 (0.53-2.10) | 0.874 |  |  |
| ≥60 years | 1.63 (0.83-3.20) | 0.156 |  |  |
| Sex |  |  |  |  |
| Female | 1 |  | 1 |  |
| Male | 1.76 (1.07-2.90) | **0.027** | 3.03 (1.42-6.49) | **0.004** |
| Size (per 1cm increase) | 1.19 (1.10-1.28) | **<0.001** | 0.90 (0.71-1.14) | 0.369 |
| Size group |  |  |  |  |
| ≤5cm | 1 |  | 1 |  |
| ＞5cm | 3.08 (1.79-5.31) | **<0.001** | 1.92 (0.65-5.68) | 0.239 |
| Comorbidity |  | **0.011** |  | 0.119 |
| No Comorbidity | 1 |  | 1 |  |
| Myasthenia Gravis | 0.50 (0.24-1.07) | 0.073 | 0.67 (0.22-2.00) | 0.469 |
| Other autoimmune diseases | 2.49 (1.12-5.53) | **0.025** | 7.09 (2.29-22.01) | **0.001** |
| Pathologic type |  | **<0.001** |  | **<0.001** |
| Thymoma | 1 |  | 1 |  |
| Thymic cancer | 7.20 (4.32-12.03) | **<0.001** | 5.71 (2.22-14.71) | **<0.001** |
| Thymic neuroendocrine carcinoma | 0.00 (0.00-Inf) | 0.997 | 0.00 (0.00-Inf) | 0.997 |
| R0 resection status |  |  |  |  |
| Yes | 1 |  | 1 |  |
| No | 21.38 (12.17-37.56) | **<0.001** | 4.68 (1.02-21.47) | **0.047** |
| Surgery approach |  |  |  |  |
| Minimally invasive | 1 |  | 1 |  |
| Open surgery | 2.66 (1.42-4.95) | **0.002** | 0.96 (0.39-2.39) | 0.934 |
| Refined TNM9 staging system |  | **<0.001** |  | **0.003** |
| I | 1 |  | 1 |  |
| II | 24.73 (9.32-65.60) | **<0.001** | 5.73 (2.31-14.20) | **<0.001** |
| IIIA | 51.42 (17.20-153.66) | **<0.001** | 3.02 (0.49-18.73) | 0.234 |
| IIIB | 69.76 (18.68-260.49) | **<0.001** | 12.36 (2.13-71.52) | **0.005** |
| IVA | 146.39 (50.26-426.38) | **<0.001** | 3.75 (0.40-35.46) | 0.249 |
| IVB | 170.66 (61.50-473.56) | **<0.001** | 12.80 (2.45-66.89) | **0.003** |
| Radiotherapy |  |  |  |  |
| No | 1 |  | 1 |  |
| Yes | 2.64 (1.60-4.36) | **<0.001** | 0.31 (0.13-0.74) | **0.009** |
| Chemotherapy |  |  |  |  |
| No | 1 |  | 1 |  |
| Yes | 9.60 (5.88-15.67) | **<0.001** | 0.24 (0.07-0.88) | **0.031** |

**Supplementary Table 4 Multivariable Cox regression analysis of progression-free survival incorporating the refined TNM9 staging system^*^**

**Model performance metrics: AIC^#^ = 401.24, likelihood ratio test P < 0.001, C‑index = 0.872 (95% CI^#^: 0.778–0.967), time‑dependent AUCs at 24, 60, and 120 months were 0.990, 0.887, and 0.815, respectively, and PH^#^ assumption test P = 0.300.*

*#Abbreviations: AIC, Akaike information criterion; CI, confidence interval; AUC, area under curve; PH, proportional hazards.*
